# Effect of 10% Strontium Chloride and 5% Potassium Nitrate with Fluoride on Bleached Bovine Enamel

**DOI:** 10.2174/1874210601711010476

**Published:** 2017-08-31

**Authors:** Cristiane de Melo Alencar, Victor Feliz Pedrinha, Jesuína Lamartine Nogueira Araújo, Renata Antunes Esteves, Ana Daniela Silva da Silveira, Cecy Martins Silva

**Affiliations:** Faculty of Dentistry, Federal University of Pará, Pará, Brazil

**Keywords:** Tooth bleaching, Dental enamel, Dentin desensitizing agents, Strontium, Tooth remineralization, 5% Potassium nitrate

## Abstract

**Background:**

Dental whitening has been increasingly sought out to improve dental aesthetics, but may cause chemical and morphological changes in dental enamel surfaces.

**Objective:**

This study evaluated **in vitro** the effect of 10% strontium chloride and 5% potassium nitrate with fluoride on bovine enamel, through tristimulus colorimetry, Knoop microhardness (KHN), and roughness after bleaching with 35% hydrogen peroxide (HP).

**Methods:**

The specimens were divided into three groups (n=15): GControl received bleaching treatment with 35% HP; GNitrate received bleaching with 35% HP followed by the application of 5% potassium nitrate with 2% sodium fluoride; and GStrontium received bleaching with 35% HP followed by the application of 10% strontium chloride on the enamel. Next, five specimens of each experimental group were subjected to KHN and tristimulus colorimetry tests, and 10 specimens were subjected to surface roughness (SR) tests. The values obtained for the different groups were compared through analysis of variance (ANOVA) followed by a post-hoc Tukey-Kramer test in addition to Student’s T-test for paired data.

**Results:**

In the intergroup comparison, KHN final differed statistically (*p*<0.05). The mean SR final of the experimental groups differed statistically from the GControl group (*p*<0.05). In addition, the groups did not differ in color variation (*p*>0.05).

**Conclusion:**

10% strontium chloride and 5% potassium nitrate combined with 2% fluoride downplayed morphological changes to the enamel, without interfering with the effectiveness of the bleaching process.

## 1 INTRODUCTION

In modern Western culture, physical appearance is of the utmost importance [1]. The current beauty standard is well-contoured and well-aligned white teeth. Beauty is a determining factor in self-esteem that, in turn, is crucial in interpersonal relationships [2]. Changes in appearance may affect human psychological and social behavior [3]. Hydrogen peroxide (HP) is a thermally unstable chemical agent with high oxidative power, which dissociates into free radicals and reactive oxygen species, such as superoxide anion, and hydroxyl and perhydroxyl radicals [4, 5]. Being highly reactive, these molecules quickly cross the dentin-enamel junction reaching the underlying dentin and through organic reactions cause the bleaching effect [6].

However, multiple side effects related to bleaching have been found to occur on the dental structure and different restorative materials, including increased roughness, decreased hardness, and changes in surface morphology [7]. These morphological changes and the decrease in hardness are not limited to the enamel surface and have also been detected in the enamel subsurface [8].

Bistey *et al.* observed a significant reduction in the enamel fluoride content after bleaching with HP at both low and high concentrations [9]. Some authors have suggested the addition of fluoride to bleaching gels with the aim of reducing changes that occur in the enamel after bleaching and preserving the maximum strength of the enamel [10]. Kwon *et al.* (2002) reported that the pores formed on the enamel surface after bleaching occur due to the rupture of the enamel protein matrix and subsequent loss of crystalline material surrounded by this matrix [11]. This hypothesis arose from the observation in various studies that enamel dissolution occurs heterogeneously, with areas of erosion interspersed with areas of intact enamel [12-15].

Aiming to minimize the deleterious effects of dental bleaching, there has been an increase in the use of insoluble materials that precipitate on the dental surface or that facilitate the formation of biological natural minerals. These substances have shown to be effective in sealing open dentinal tubules and, consequently, in reducing the morphological changes in the enamel after bleaching [16, 17].

Strontium chloride, for example, was the first bioactive material added to toothpaste for sealing dentinal tubules, approximately 50 years ago [17]. It has been reported that strontium chloride acts through the precipitation of particles on the dental surface, preventing the movement of fluids [18]. The strontium salts can replace the calcium in hydroxyapatite due to the chemical similarity of these elements, favoring tissue remineralization and sealing open dentinal tubules [19].

Another current and widely used bioactive material is potassium nitrate combined with sodium fluoride. The use of fluoride-based materials at low concentrations has been recommended for reestablishing or protecting the structure of dental tissues [20, 21]. The remineralization of enamel through the use of sodium fluoride is characterized by the induction of fluorapatite or fluorhydroxyapatite formation through direct or indirect reactions with hydroxyapatite, thereby transforming the phosphate calcium present in the oral cavity. The fluoride ions must be freely accessible in the oral medium for better efficiency in the enamel remineralization process to be obtained [22].

This study aims to evaluate, *in vitro*, the effect of strontium chloride and potassium nitrate combined with fluoride on the hardness, morphological changes, and color of bovine enamel bleached with 35% HP through an assessment of Knoop microhardness (KHN), surface roughness (SR), and colorimetry.

## 2 MATERIALS AND METHODS

### 2.1 Bioethics Committee

This study was approved by the Animal Research Ethics Committee of the Federal University of Pará through number 026-2015.

### 2.2 Selection and Storage of Teeth

Bovine incisors donated by the Cooperativa da Indústria Pecuária do Pará – SOCIPE, in the city of Belém, state of Pará (PA), Brazil, were used. The teeth were collected following a protocol approved by the committee of the Federal University of Pará. Sixty-five intact and erupted bovine incisors were extracted immediately after the animals were sacrificed. The teeth were cleaned with the use of Gracey curettes (Trinity Indústria e comércio LTDA, Jaraguá, São Paulo, Brazil) to remove possible tartar and then subjected to prophylaxis using a Robson brush (Flat Prophy Brush – RA – White, Microdont, Socorro, São Paulo, Brazil) and prophylactic toothpaste (Pasta Profilática Odahcam, Dentsply, Petrópolis, RJ, Brazil) for total removal of organic and inorganic debris from the coronal surface. Each tooth was sectioned cross-sectionally at the crown/root interface along the dentin-enamel junction, with the aid of a double-sided diamond disk (KG Sorensen, São Paulo, Brazil) under air/water cooling; the crowns were used for the experiment, and the roots were discarded. Then, the samples were stored in distilled water at room temperature until specimen preparation.

### 2.3 Specimen Preparation

Only the vestibular surfaces of the crowns were used for the study. The vestibular surface was sectioned for obtaining a single fragment of its central region, using a double-sided diamond disk (Microdont, Socorro, São Paulo, Brazil), fitted to a low-speed micromotor (Micromotor 500-Kavo, Joinville, Santa Catarina, Brazil) under cooling with running water to avoid burning the dental tissue. The enamel surfaces of the teeth were planned and polished using #600, #1200, and #2000 water sandpaper in a polishing machine (AROTEC LMTD, São Paulo, Brazil), removing only enough material to obtain a flat surface, without any scratches or irregularities, for performing the microhardness and roughness readings. The fragments were washed with distilled water to remove any possible debris from the surface of the fragment. After preparation, the fragments were stored in distilled water until the beginning of the bleaching treatment.

### 2.4 Experimental Groups

**Fig. (1):**
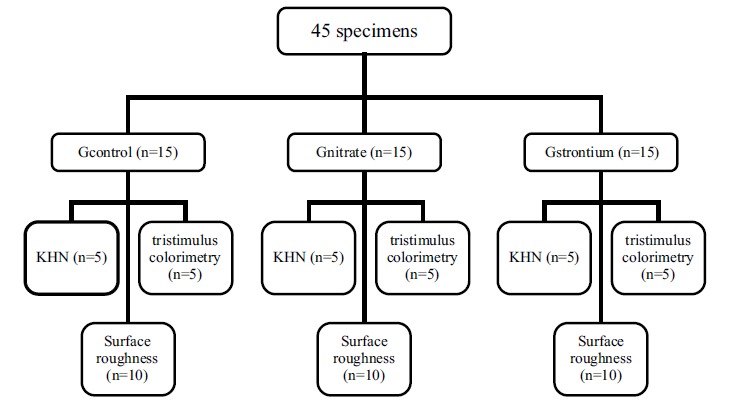
Experimental draw.

Forty-five specimens were divided into three groups (n=15): group 1 (GControl), consisting of specimens receiving only the bleaching treatment with 35% HP (White and Brite Advanced, 3M/ESPE, Sumaré, São Paulo, Brazil); group 2 (GNitrate), consisting of specimens bleached with 35% HP (White and Brite Advanced, 3M/ESPE) followed by application of 5% potassium nitrate combined with 2% sodium fluoride (Desensibilize KF 2% (2.5g)-FGM/ Joinville, SC, Brazil) on the enamel surface for five minutes; group 3 (GStrontium), consisting of specimens bleached with 35% HP (White and Brite Advanced, 3M/ESPE, Sumare, São Paulo, Brazil) followed by application of 10% strontium chloride (Sensodyne Original^®^ (50g)-Glaxo Smith Kline/Rio de Janeiro, RJ, Brazil) on the enamel surface for five minutes. Then, five specimens of each experimental group were subjected to KHN and tristimulus colorimetry tests, and 10 specimens were subjected to SR tests (Fig. **1**).

### 2.5 Dental Bleaching + Desensitizing Agent

The application of bleaching agents followed the recommendations of the manufacturer. The specimens received three applications of 15 minutes per session, totaling 45 minutes. Four sessions were conducted at seven-day intervals. Then, the specimens were cleaned and rinsed in running water and then underwent surface polishing with polishing paste (Diamond Excel, FGM/Joinville, SC, Brazil) using a felt wheel (Diamond Flex, FGM/Joinville, SC, Brazil) attached to a low-speed handpiece (Dabi-Atlante/ Ribeirão Preto, SP, Brazil). GNitrate and GStrontium received 10% strontium chloride and 2% potassium nitrate, respectively, for five minutes after polishing in each treatment session. Between treatment sessions, the specimens were stored in artificial saliva in a biological greenhouse at 37 °C (Q316M2 – 1’3/288 V – 188W, Quimis* Aparelhos Científicos LTDA, São Paulo, Brazil). The artificial saliva (potassium chloride, sodium chloride, magnesium chloride, potassium phosphate, calcium chloride, preservatives, carboxymethyl cellulose, sorbitol; aqueous solution q.s.; Pharmacy Apis Mel, Belém, Pa, Brazil) was renewed daily.

### 2.6 Knoop Microhardness

Five specimens were used for each group, on which five indentations were made, which were separated by 100 µm in each specimen using a load of 25 gf for five seconds in the microhardness tester (FM-700, Future Tech Corp., Tokyo, Japan). The location of the indentations for making the readings was such that it was possible to hypothetically map the total area of the specimens (left and right ends, top and bottom, and center). Readings of KHNinitial (before bleaching treatment) and KHNfinal (immediately after the end of the bleaching treatment) were obtained.

The percent change in the KHN (%KHN) was calculated using the formula:

%KHN=KHNfinal−KHNinitial∗100KHNinitial

### 2.7 Surface Roughness

Ten specimens were used for the SR reading. A roughness meter (Surftest Mitsutoyo South American LTDA, São Paulo, Brazil) was used to measure SR before and after the bleaching procedures. The tip of the roughness meter touched the specimen and explored the central 4 mm, making three measures diametrically opposed for obtaining the mean roughness (Ra), which was previously established and calibrated using the device’s software. The changes in the anatomical surface of the specimen (plane or convex) were compensated for by the software, without affecting the roughness results.

Ra, which is the arithmetic mean of deviations from the roughness profile, was obtained by the arithmetic mean of the sum of the absolute values of the roughness profile deviations from the central line for the path evaluated.

As complementary analysis, the percent change of surface roughness (%SR) for each group was calculated according to the formula:

%SR=SRfinal−srinitial∗100SRinitial

### 2.8 Tristimulus Colorimetry Tests

The color of the specimens was assessed with the aid of a CR-400 - Konica Minolt colorimeter (Tecnal Indústria - Equipamentos para Laboratório / Piracicaba, SP) using the CIE L*a*b* system. The specimens were placed on a black surface so that the reflected light did not interfere with the color values of the specimens. To evaluate the color change between the initial and final results, ∆E was calculated using the following formula: = {∆E(∆L)2+(∆a)2+(∆b)2}1/2, where ∆L=L-L0, a=a-a0, and ∆b=b-b0. ΔL, ∆a, and Δb represent the variation in the black-white, green-red, and yellow-blue coordinates, respectively.

### 2.9 Statistical Analysis

The KHN, roughness, and color (∆E) values were tabulated on an Excel spreadsheet (Microsoft Windows 2010) and analyzed using the software BioEstat^®^ (Sociedade Civil Mamirauá).

The normality of the variables was evaluated by the Shapiro-Wilk test. For all comparisons between the groups, analysis of variance (ANOVA) was used, followed by the post-hoc Tukey-Kramer test. For the comparison between the experimental times within the same group, Student’s t-test for paired data was used. For all analyses, a significance level of 5% was considered.

## 3 RESULTS

### 3.1 Knoop Microhardness

Fig. (**2**) shows the mean KHN values. Student’s t-test (*p*<0.05) revealed in the intergroup comparison between the mean KHNinitial of the evaluated groups did not differ statistically *(p*>0.05). However, the post-hoc Tukey-Kramer test revealed in the intergroup comparative analysis that KHNfinal showed a statistically significant difference (*p*<0.05). GControl presented the lowest mean KHN compared with the other groups evaluated, followed by GNitrate and GStrontium, in that order.

### 3.2 Surface Roughness

Fig. (**3**) lists the comparisons between the final and initial SR means. Based on Student’s t-test (*p*<0.05), GControl and GStrontium presented statistically significant differences between the initial and final SR means; however, GNitrate showed no significant difference. GControl presented a significant increase in the mean SR final (*p*<0.05), whereas GStrontium showed a significant decrease in the mean SR final (*p*<0.05).

The post-hoc Tukey-Kramer test (*p*>0.05) showed in the intergroup comparison that the mean SRinitial did not differ between the groups evaluated. However, the mean SRfinal of the experimental groups presented a significant difference from the control group. GNitrate and GStrontium did not differ between themselves (*p*>0.05).

### 3.3 Tristimulus Colorimetry

 Table **1** lists the post-hoc Tukey-Kramer results *(p*>0.05) and shows that the experimental groups did not statistically differ among themselves regarding color variation (ρE) (*p*=0.61).

## 4 DISCUSSION

The use of bovine teeth in this study is justified by the similarity of this substrate with the human enamel with regard to its hardness and chemical and biological composition [23]. GControl, which was only bleached with 35% HP, exhibited a reduced KHN and an increased SR of the enamel, which is in agreement with the results of previous studies [24-26].

The redox nature of bleaching agents can be used as a theoretical basis for understanding the dynamics of the bleaching process [27]. The bleaching agents suffer ionization and decomposition, producing free radicals, which are highly unstable and present a remarkable ability to react with organic substances present in the macromolecules of tooth pigments. At the saturation point, the bleaching agent acts on other compounds that have carbon chains, such as the enamel matrix proteins, causing loss of dental structure [28]. In addition, the reaction mechanism of the bleaching agent and its deleterious effects may explain the significant morphological changes represented by the decrease in hardness and increase in SR observed in GControl.

The application of 10% strontium chloride on the bleached enamel (GStrontium) not only minimized the deleterious effects of HP but also promoted a significant increase in hardness and a decrease in SR. It is likely that these effects are due to the remineralizing properties of this bioactive material.

*In vitro* studies have reported that strontium synergistically reacts with the fluoride present in the oral cavity and is thereby associated with an improvement in the mineral density of the demineralized dentine. Furthermore, the strontium salts can deposit a layer of thin particles, sealing and penetrating the dentinal tubules [29-31].

Smaller morphological changes were observed in GNitrate (5% potassium nitrate and 2% sodium fluoride). It is likely that the prevention of demineralizing effects promoted by dental bleaching is due to the remineralizing action of 2% sodium fluoride [32]. Fluoride, when in contact with dental element, leads to the formation of calcium fluoride deposits on the enamel, dentine, and cement surfaces. These deposits act as a fluoride reservoir in the oral cavity to be used in the processes of demineralization and remineralization. Therefore, enamel remineralization using sodium fluoride is characterized by the induction of fluorapatite formation. In addition, the fluoride action would explain the decrease in SR in this experimental group [33, 34].

However, 5% potassium nitrate and 2% sodium fluoride did not promote a significant increase in the surface microhardness compared with the group that used 10% strontium chloride. This result might be due to the active properties of potassium ions, which are the main active components in the dental-sensitivity preventive process in vital teeth, reducing dentinal sensory nerve activity caused by depolarization produced by the painful stimulus [35-37]. Therefore, such effects could be better observed in clinical or *in vivo* studies on dentinal sensitivity.

A colorimeter, which is considered accurate for measuring color, was used to evaluate the effectiveness of the bleaching treatment. Colorimetry is a quantitative method for evaluating color that allows for identifying color variation [38]. This color evaluation method uses the standardization of the Commission Internationale l' Eclairage, in which the values of the L *, a *, and b * coordinates of each tooth, referring to the variation in black and white (luminosity), red-green, and blue-yellow, respectively, are evaluated, enabling calculation of the total color variation (∆E). A ∆E of 3.3 to 3.7 becomes clinically noticeable after dental bleaching [39].

The ∆E results obtained in the present study showed that the experimental groups had a similar behavior to that of the control group during the evaluation periods. The use of 10% strontium chloride and 5% potassium nitrate combined with the bleaching treatment, despite its remineralizing effects, did not affect the penetration of peroxide molecules, and consequently, there was no impairment of the dental bleaching process.

Although the experimental formulations and treatment time have shown satisfactory results, clinical and *in vivo* experiments that assess the effectiveness of the bioactive materials investigated in this study are of utmost importance. Thus, further studies that can contribute to the prevention of deleterious effects from bleaching treatments must be performed.

## CONCLUSION

Based on the methodology employed in this study, it was concluded that the use of 10% strontium chloride and 5% potassium nitrate combined with 2% fluoride downplayed changes in the microhardness and SR of bovine enamel bleached with 35% HP without affecting the effectiveness of the bleaching treatment.

## Figures and Tables

**Fig. (2):**
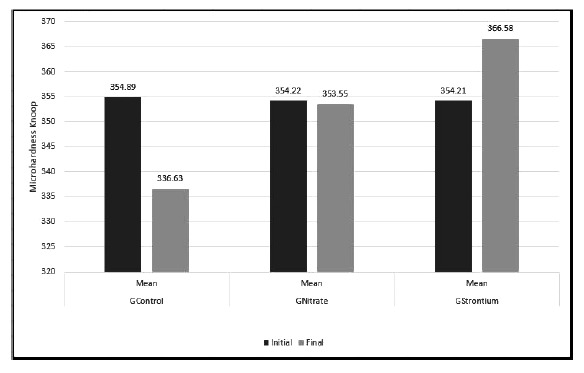
Means, standard deviations of microhardness Knoop start and end of the study groups.

**Fig. (3):**
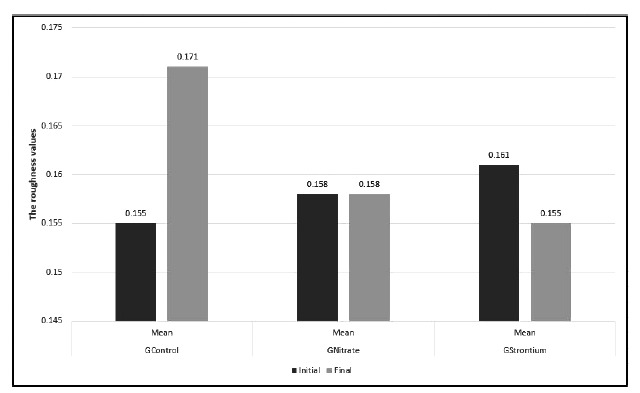
Mens, standard deviations the roughness values start and end of the study groups.

**Table 1:** Means, standard deviations and confidence interval (CI) (95%) of ΔE values for each of the experimental groups.

	**GControl**	**GNitrate**	**GStrontium**	***p***
(±DP)	11.82 (±0.48)	11.72 (±0.68)	11.46 (±0.56)	0.61
CI (95%)	10.87 – 12.77	10.37 – 13.07	10.34 – 12.58	
